# Higher Expression of Receptor Tyrosine Kinase Axl, and Differential Expression of its Ligand, Gas6, Predict Poor Survival in Lung Adenocarcinoma Patients

**DOI:** 10.1245/s10434-012-2795-3

**Published:** 2012-12-16

**Authors:** Masashi Ishikawa, Makoto Sonobe, Ei Nakayama, Masashi Kobayashi, Ryutaro Kikuchi, Jiro Kitamura, Naoto Imamura, Hiroshi Date

**Affiliations:** Department of Thoracic Surgery, Faculty of Medicine, Kyoto University, Kyoto, Japan

## Abstract

**Background:**

Downstream activation through receptor tyrosine kinases (RTKs) plays important roles in carcinogenesis. In this study, we assessed the clinical involvement of Axl, an RTK, and its ligand, Gas6, in surgically treated lung adenocarcinoma.

**Methods:**

*Axl* and *Gas6* mRNA and protein expression levels were quantified using quantitative real-time polymerase chain reaction and immunohistochemistry, respectively, in completely resected lung adenocarcinoma tissues (*n* = 88) and were evaluated for correlation with clinicopathologic features and patient survival.

**Results:**

Higher expressions of Axl mRNA/protein and Gas6 protein were significantly related to worse clinicopathological features and prognosis (5-year overall survival rates: *Axl* mRNA low: 72.3 %, high: 49.7 %, *P* = 0.047; Axl protein low: 77.5 %, high: 38.6 %, *P* < 0.001; and Gas6 protein low: 70.5 %, high: 48 %, *P* = 0.042). On the contrary, higher *Gas6* mRNA expression was related to better clinicopathological features and prognosis (5-year overall survival rates: *Gas6* mRNA low: 59.2 %, high: 81.8 %, *P* = 0.054). Multivariate analysis suggests that high *Axl* mRNA expression may be an independent factor for poor patient prognosis (*P* = 0.04).

**Conclusions:**

In lung adenocarcinoma, Axl and Gas6 expression levels were associated with tumor advancement and patient survival, thus rendering them as reliable biomarkers and potential targets for treatment of lung adenocarcinoma.

Non-small cell lung cancer (NSCLC) has been the leading cause of cancer-related deaths world-wide, and adenocarcinoma accounts for about half of all NSCLC cases.^1^,^2^ Recent strategies for cancer treatment have focused on inhibiting oncogenic pathways in specific cancers. Receptor tyrosine kinases (RTKs) are some of the most common classes of molecules investigated for that purpose, and in the treatment of lung adenocarcinoma, disrupting them with small-molecule RTK inhibitors (TKIs) have already provided us with new and preferred treatment options.^3^,^4^ Unfortunately though, the targeted patient population and therapeutic effects are still limited, thus further investigation on RTKs is vital to provide more therapeutic alternatives.

Recently, the TAM family of RTKs has been reported to regulate various biological processes.^5^ This family consists of Tyro3 and Mer as well as Axl.^6^ Axl has already been reported to be overexpressed in several human cancers.^7^–^11^ Its natural ligand, Gas6, binds Axl with three- to tenfold higher affinity than Mer and causes Axl phosphorylation, which results in activation of various downstream pathways.^12^,^13^ Thus, Axl/Gas6 signaling contributes to a variety of oncogenic mechanisms.^14^,^15^

Although some reports have already mentioned the expression or effects of Axl in lung cancer, specific or detailed clinical impact of both Axl and Gas6 expression have not, thus far, been fully investigated.^16^,^17^

In this study, we retrospectively evaluated Axl and Gas6 expression levels in lung adenocarcinoma at both the genetic and protein levels, confirming their role in carcinogenesis, which we believe will be useful clinical reference in utilizing these molecules as cancer biomarkers and future therapeutic targets for the treatment of lung cancer.

## Materials and Methods

### Patients, Tissue Samples, and Tumor Information

Tissue samples were obtained from patients who had undergone complete surgical resection of primary lung adenocarcinoma without any prior anticancer therapies at Kyoto University Hospital between 2001 and 2005 (*n* = 88). All of these tumors were histologically confirmed as lung adenocarcinoma, and their lymph node (LN) metastatic status, pathological (p-) staging, degree of differentiation, and pleural/vascular/lymphatic invasion status were all assessed by board-certified pathologists in the Department of Pathology of Kyoto University Hospital. P-stage was determined by the latest tumor-node-metastasis classification system.^18^ Histological type and grade of cell differentiation were determined according to the WHO classification system.^19^ *EGFR* gene mutations (exons 18-21) were detected using polymerase chain reaction (PCR) single-strand conformational polymorphism analysis, and *K*-*ras* gene mutations (codon 12) were screened using the mutagenic PCR restriction enzyme fragment length polymorphism method.^20^ Informed consent for participation in this study was obtained from all patients before their surgeries, and this study was reviewed and approved by the Ethics Committee of the Graduate School and Faculty of Medicine at Kyoto University.

### Preparation of Tissue mRNA

For sample collection, tumor tissue samples were dissected immediately after surgical resection and soaked in RNAlater TissueProtect Tubes (Qiagen, Tokyo, Japan) for more than 48 h before storage at −80 °C until use. Total RNA was isolated from tissue samples using RNeasy Plus Mini Kit (Qiagen), and reverse transcription of total RNA was conducted using the Ready-To-Go You-Prime First-Strand Beads (Amersham Biosciences, Uppsala, Sweden) to obtain cDNA.

### Quantification and Evaluation of Axl and Gas6 mRNA

To quantify *Axl* and *Gas6* mRNA expression levels of each sample, quantitative real-time PCR was performed using the LightCycler thermal cycler system (Roche Diagnostics Japan, Tokyo, Japan). The PCR primers used for the quantitative amplification of *Axl* mRNA were forward: 5′-GGTGGCTGTGAAGACGATGA-3′ and reverse: 5′-CTCAGATACTCCATGCCACT-3′, and those of *Gas6* mRNA were forward: 5′-ACATCTTGCCGTGCGTGCCCTTCA-3′ and reverse: 5′-ATTCCGCGCCAGCTCCTCAACAGA-3′. The primers for *glyceraldehyde*-*3*-*phosphate dehydrogenase* (*GAPDH*) mRNA, used as an internal control, were forward: 5′-ACAACAGCCTCAAGATCATCAG-3′ and reverse: 5′-TCTTCTGGGTGGCAGTGATG-3′. After a 20-μL reaction mixture containing 0.5 μM forward and reverse primers and 0.03 μg cDNA in QuantiTect SYBR Green PCR Master Mix (Qiagen) was prepared, PCR amplification was initiated by preincubation for 15 min at 95 °C for initial activation, followed by 40 cycles of the following protocol: denaturation at 94 °C for 15 s (sec), annealing at 59 °C for 15 s, and elongation at 72 °C for 15 s with detection of fluorescence products. The quantitative data were analyzed with LightCycler analysis software version 5.03 (Roche Diagnostics Japan). The expression levels of *Axl* and *Gas6* were represented as the ratio of *Axl* or *Gas6* mRNA value to *GAPDH* mRNA value. The patients were dichotomized on the basis of the mean value of *Axl* or *Gas6* mRNA expression, and their clinicopathologic features and survival curves were later analyzed.

### Immunohistochemistry of Axl and Gas6

Immunohistochemical (IHC) staining was performed using Dako LSAB + System-HRP (Dako Japan, Tokyo, Japan). Formalin-fixed, paraffin-embedded tissue was cut into 4-μm sections and mounted on glass slides. After deparaffinization and rehydration, the slides were heated in a buffer solution (HistoVT One, Nacalai Tesque, Kyoto, Japan) for antigen retrieval at 90 °C for 20 min. After quenching the endogenous activity with 0.3 % hydrogen peroxide (in absolute methanol) for 10 min, the sections were treated with blocking agent (DAKOCytomation Protein Block, Dako Japan) for 30 min to block nonspecific staining. The sections were incubated overnight with a rabbit anti-Axl polyclonal antibody (sc-20741, 1:100, Santa Cruz Biotechnology, Inc., CA, USA) or a goat anti-human polyclonal Gas6 antibody (AF885, 1:100, R&D Systems Inc., MN, USA). The slides were then incubated for 50 min each with the secondary antibody (Biotinylated Link, Dako Japan) and peroxidase (STREPTOAVIDIN-HRP, Dako Japan), followed by visualization with 3,3′-diaminobenzine tetrahydrochloride (DAB + CHROMOGEN, Dako Japan). Finally, the sections were counterstained with Mayer’s hematoxylin (Dako REAL Hematoxylin, Dako Japan). The negative control slides were prepared by replacing the primary antibody with an irrelevant mouse immunoglobulin G (N1698, Dako Japan).

### Evaluation of IHC Results

Axl and Gas6 protein expression were estimated according to a semiquantitative scoring system, in which the staining intensity was graded as 0 (no staining), 1 (weak), 2 (moderate), or 3 (strong), and percentage of positive cells was graded as 0 (negative), 1 (≤10 %), 2 (11–50 %), 3 (51–80 %), or 4 (>80 %). The final IHC score was obtained by multiplication of both grading results (staining intensity × percentage of positive cells). The IHC scores were compared within each clinical category, and the clinicopathological features and survival curves were analyzed after dichotomization according to their staining intensity (each IHC score: ≤7 vs. >7).

### Statistical Analysis

Statistically significant differences within categorical data were determined using the χ^2^ test. Continuous data of two groups were compared using Student’s *t* test and that of three or more groups were compared using ANOVA. Survival curves were evaluated with the Kaplan–Meier method, and cumulative survival data were compared using the log-rank test. Multivariate analysis of prognostic factors was performed by the Cox proportional hazard model. Differences were considered significant when *P* < 0.05. mRNA expression levels and IHC scores are expressed as median and mean ± standard deviation. All statistical analyses were performed using StatMate IV software version 4.01 (ATMS, Tokyo, Japan) and JMP software version 8 (SAS Institute Japan, Tokyo, Japan).

## Results

### Axl and Gas6 mRNA Expression Correlated well with Clinical Variables

The level of *Axl* mRNA (normalized and expressed as a ratio to *GAPDH* mRNA) ranged from 0 to 8.77 × 10^−3^ (8.37 × 10^−4^, 1.39 × 10^−3^ ± 1.62 × 10^−3^), and that of *Gas6* mRNA ranged from 1.26 × 10^−4^ to 2.11 × 10^−1^ (7.59 × 10^−3^, 1.37 × 10^−2^ ± 2.68 × 10^−2^). Sixty patients were placed in the *Axl* mRNA low group and 28 patients in the high group, whereas 66 patients were placed in the *Gas6* mRNA low group and 22 patients in the high group (below versus above each mean value; Table 1).

**Table 1:** Axl/Gas6 mRNA and protein expressions and characteristics of the patients

	*Axl* mRNA (n = 88)		*P*	*Gas6* mRNA (n = 88)		*P*	Axl IHC score (n = 88)		*P*	Gas6 IHC score (n = 88)		*P*
	Low (n = 60)	High (n = 28)		Low (n = 66)	High (n = 22)		Low (n = 59)	High (n = 29)		Low (n = 66)	High (n = 22)	
Age (yr)												
Mean ± SD (range)	65.2 ± 10.1 (45–88)	63.1 ± 11.4 (31–79)	0.39	63.4 ± 10.8 (31–88)	68 ± 8.9 (51–79)	0.074	66.3 ± 10 (43–88)	60.9 ± 10.8 (31–79)	**0.022**	64.8 ± 11 (31–88)	63.9 ± 9.07 (45–76)	0.73
Gender												
Male	35	18	0.6	42	11	0.26	32	8	**0.018**	40	13	0.9
Female	25	10		24	11		27	21		26	9	
Smoking status												
Never smoked	19	11	0.66	19	11	0.18	22	8	0.21	25	5	0.075
Ex-smoker	18	6		20	4		18	6		20	4	
Current smoker	23	11		27	7		19	15		21	13	
Pack-year/mean ± SD (range)	38 ± 43.2 (0–165)	34.2 ± 44.1 (0–180)	0.7	40.2 ± 44.7 (0–180)	26.4 ± 37.9 (0–120)	0.2	33.9 ± 42.2 (0–165)	42.6 ± 45.7 (0–180)	0.35	32.3 ± 40.5 (0–165)	49.4 ± 49.6 (0–180)	0.11
Tumor differentiation												
Well	18	12	0.41	15	15	**<0.001**	23	7	0.12	25	5	0.12
Moderately	25	11		30	6		25	11		28	8	
Poorly	17	5		21	1		11	11		13	9	
P-stage												
IA	26	11	0.069	22	15	**0.018**	33	4	**<0.001**	33	4	0.053
IB	14	3		13	4		13	4		12	5	
IIA	6	0		5	1		3	3		3	3	
IIB	1	1		1	1		2	0		2	0	
IIIA	13	13		25	1		8	18		16	10	
Maximum tumor size (mm)												
Mean ± SD (range)	25.9 ± 10.5 (7–50)	25.4 ± 13.7 (10–68)	0.86	27.3 ± 11.8 (10–68)	21.0 ± 9.6 (7–40)	**0.026**	24.7 ± 11.2 (7–68)	27.7 ± 12.2 (10–55)	0.26	23.8 ± 9.6 (7–50)	31.3 ± 15 (13–68)	**0.008**
Lymph node metastatic status												
n0	40	14	0.22	35	19	**0.013**	46	8	**<0.001**	45	9	**0.045**
n1	7	3		8	2		6	4		5	5	
n2	13	11		23	1		7	17		16	8	
Pleural invasion status												
p0	43	16	**0.013**	42	17	0.73	43	16	0.41	45	14	0.21
p1	8	2		8	2		6	4		7	3	
p2	8	3		9	2		6	6		10	1	
p3	1	6		6	1		3	3		3	4	
Unknown	0	1		1	0		1	0		1	0	
Vascular invasion status												
Negative	28	21	0.12	36	13	0.061	28	21	0.48	37	12	0.25
Positive	14	4		17	1		12	6		11	7	
Unknown	18	3		13	8		19	2		18	3	
Lymphatic invasion status												
Negative	32	20	0.3	38	14	**0.035**	33	19	0.25	39	13	0.33
Positive	10	3		13	0		6	7		8	5	
Unknown	18	5		15	8		20	3		19	4	
*EGFR* gene mutation status												
Wild-type	36	13	0.23	42	7	**0.0093**	33	16	0.95	34	15	0.17
Mutation (+)	24	15		24	15		26	13		32	7	
*K*-*ras* gene mutation status												
Wild-type	50	25	0.46	58	17	0.22	48	27	0.14	56	19	0.86
Mutation (+)	10	3		8	5		11	2		10	3	

Bold values are statistically significant

*SD* standard deviation

A statistically significant correlation was found between pleural invasion status and *Axl* mRNA expression. *Gas6* mRNA expression was significantly associated with tumor differentiation, p-stage, maximum tumor size, LN metastatic status, lymphatic invasion status, and *EGFR* gene mutation status, which unexpectedly showed that the high *Gas6* mRNA expression group included more patients with better clinicopathological features.

### Axl and Gas6 mRNA Expression Reflected Postoperative Patient Survival

**Fig. 1:**
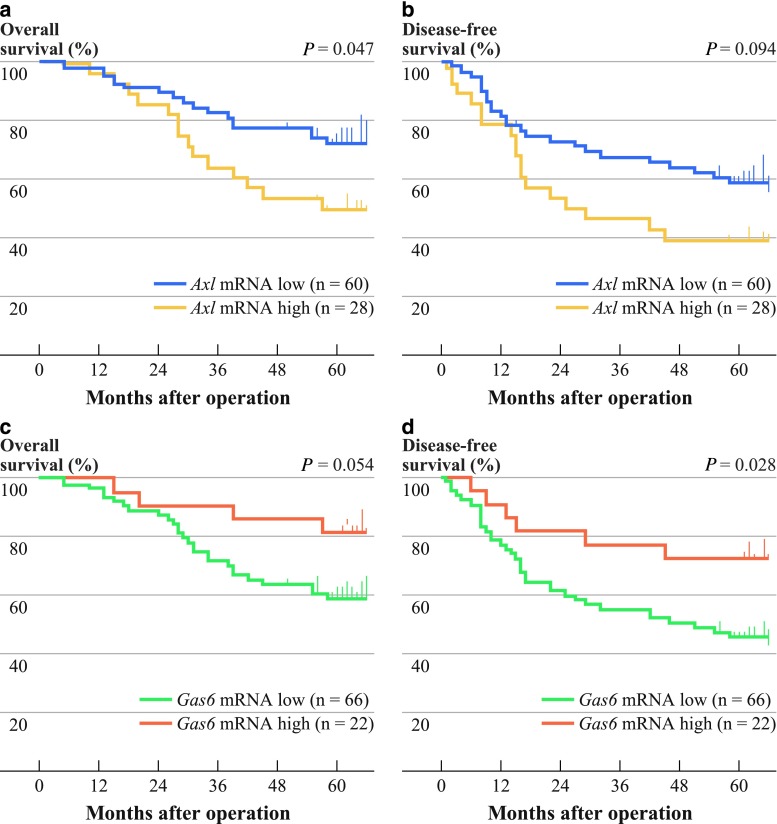
High *Axl* mRNA and low *Gas6* mRNA expressions in lung adenocarcinoma tissue correlated with poorer patient survival. Kaplan–Meier curves for overall (**a**, **c**) and disease-free (**b**, **d**) survivals according to *Axl* (**a**, **b**) and *Gas6* (**c**, **d**) mRNA expression levels. *P* values were calculated by the log-rank test. *Ticks* censored cases

Five-year overall survival (5y-OS) rates in the *Axl* mRNA low and high groups were 72.3 and 49.7 %, respectively, which was statistically significant (*P* = 0.047; hazard ratio (HR) = 0.5; 95 % confidence interval (CI), 0.21–0.99; Fig. 1a). Five-year, disease-free survival (5y-DFS) rates in the *Axl* mRNA low and high groups were 58.9 % and 39.3 %, respectively (*P* = 0.094; Fig. 1b).

Similarly, the 5y-OS rates in the *Gas6* mRNA low and high groups were 59.2 and 81.8 %, respectively (*P* = 0.054; Fig. 1c). The 5y-DFS rates in the *Gas6* mRNA low and high groups were statistically different at 45.5 and 72.7 %, respectively, and surprisingly, log-rank testing revealed significantly better patient DFS in the *Gas6* mRNA high group (*P* = 0.028; HR = 2.5; 95 % CI, 1.1–5.7; Fig. 1d).

### Axl and Gas6 Protein Expression also Correlated well with Clinical Variables

**Fig. 2:**
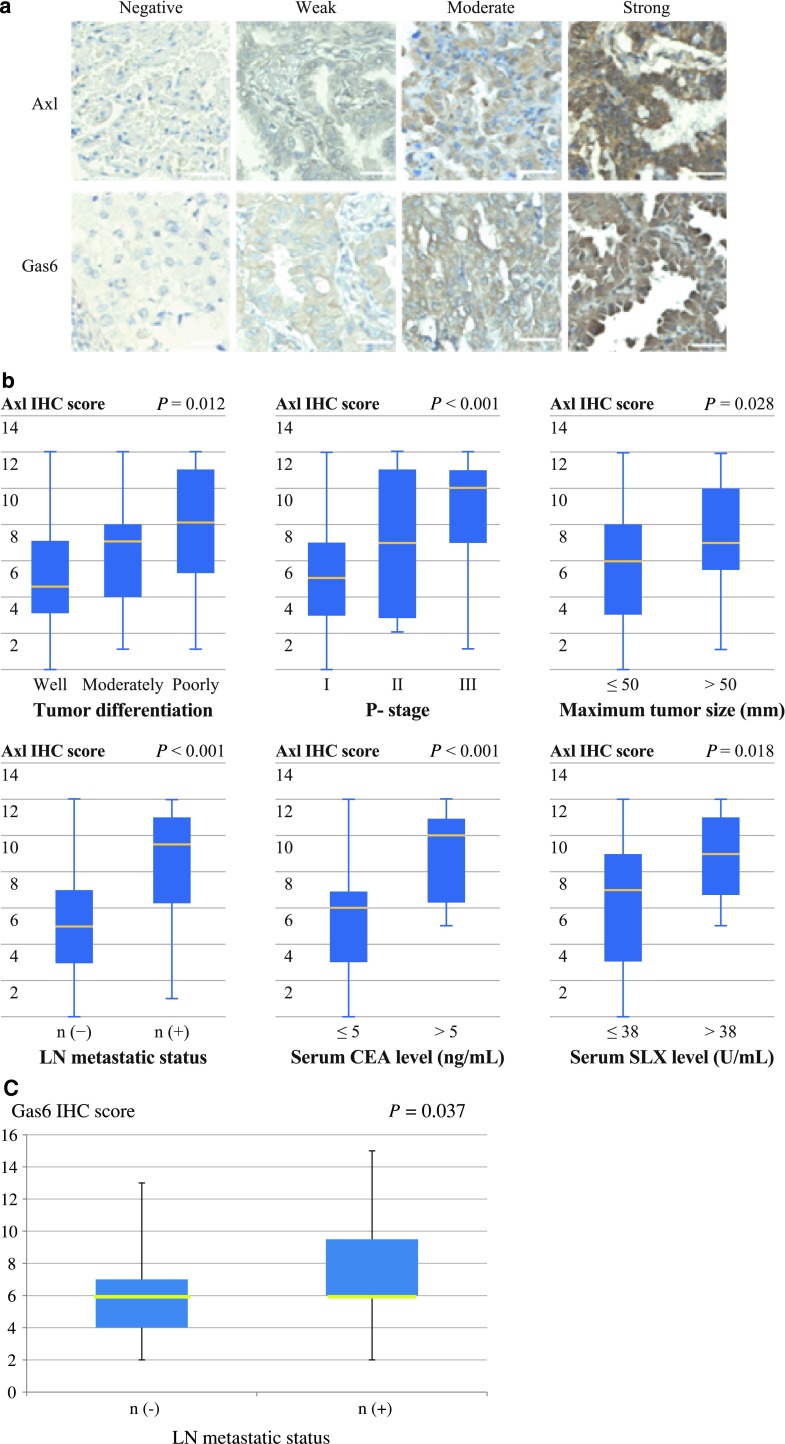
**a** Immunohistochemical staining analysis of lung adenocarcinoma tissue for Axl (*top*) and Gas6 (*bottom*), showing cases with negative, weak, moderate, and strong staining patterns. *Scale bar* 20 μm. **b** Axl immunoreactivity increased according to tumor differentiation, pathological (p-) stage, maximum tumor size, lymph node (LN) metastatic status, and serum tumor marker levels in lung adenocarcinoma tissue at time of resection. **c** Gas6 immunoreactivity also correlated well with LN metastatic status. *CEA* carcinoembryonic antigen; *SLX* sialyl Lewis X-i antigen. *P* values were calculated by Student’s *t* test or ANOVA

The IHC score for Axl ranged from 0 to 12 (7, 6.3 ± 3.3) and that of Gas6 ranged from 2 to 15 (6, 6.3 ± 2.8). Representative images of Axl and Gas6 IHC at each staining intensity level are presented in Fig. 2a. Statistically significant associations were found between Axl IHC score and tumor differentiation, p-stage, maximum tumor size, LN metastatic status, and serum tumor marker levels (Fig. 2b), as well as between Gas6 IHC score and LN metastatic status (Fig. 2c).

Fifty-nine and 29 patients were placed in the Axl IHC low and high groups, respectively, whereas 66 and 22 patients were included in the Gas6 IHC low and high groups, respectively (each IHC score: ≤7 vs. >7; Table 1).

Statistically significant differences were found between the Axl IHC low and high groups with regard to age, gender, p-stage, and LN metastatic status, and between the Gas6 IHC low and high groups with respect to maximum tumor size and LN metastatic status.

### Axl and Gas6 Protein Expression Predicted Patient Survival

**Fig. 3:**
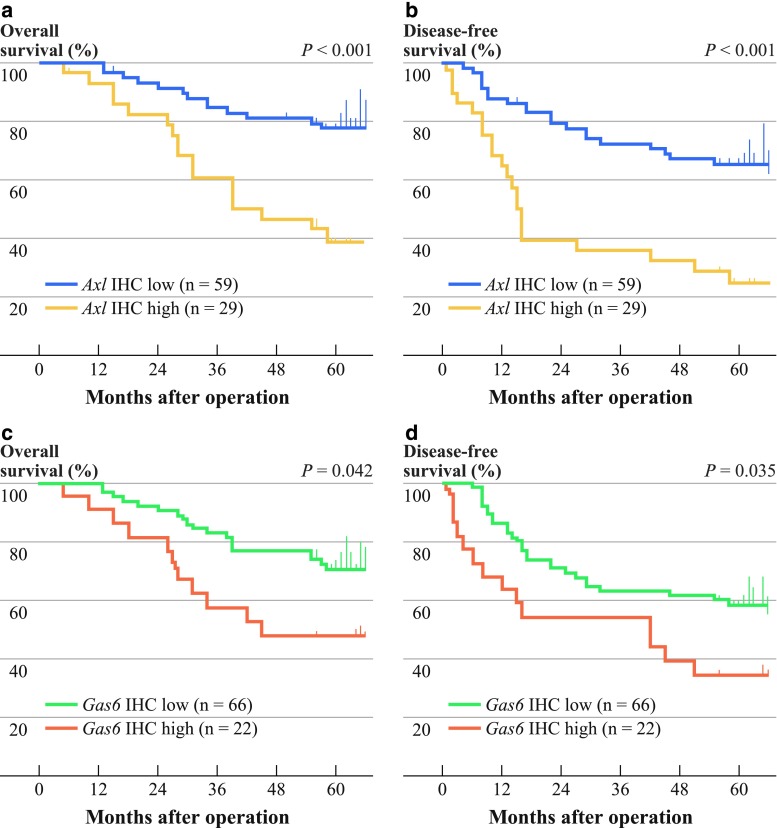
High Axl and Gas6 immunoreactivity in lung adenocarcinoma tissue predicted poorer patient survival. Kaplan–Meier curves for overall (**a**, **c**) and disease-free (**b**, **d**) survivals according to Axl (**a**, **b**) and Gas6 (**c**, **d**) immunohistochemical (IHC) scores. *P* values were calculated by the log-rank test. *Ticks* censored cases

The 5y-OS rates in the Axl IHC low and high groups were 77.5 and 38.6 %, respectively, which is statistically significant (*P* < 0.001; HR = 0.31; 95 % CI, 0.11–0.54; Fig. 3a). There also was a statistical difference in the 5y-DFS rates between the Axl IHC low and high groups at 65.7 and 24.7 %, respectively (*P* < 0.001; HR = 0.33; 95 % CI, 0.12–0.49; Fig. 3b).

Similarly, the 5y-OS rates in the Gas6 IHC low and high groups were 70.5 and 48 %, respectively (*P* = 0.042; HR = 0.48; 95 % CI, 0.17–0.97; Fig. 3c). The 5y-DFS rates in the Gas6 IHC low and high groups were 58.4 and 34.5 %, respectively, with statistical significance as well (*P* = 0.035; HR = 0.53; 95 % CI, 0.21–0.95; Fig. 3d).

### High Axl mRNA Expression may be an Independent Factor of Poor Prognosis in Surgically Treated Lung Adenocarcinomas

The results of the univariate analyses for each clinicopathological parameter and the multivariate analyses are presented in Table 2. We found that high *Axl* mRNA expression may be an independent factor for poor patient prognosis in lung adenocarcinoma (*P* = 0.04; HR = 1.9; 95 % CI, 1.03–3.5).

**Table 2:** Univariate and multivariate analyses of *Axl* mRNA expression and other clinicopathological variables for patient survival

Variables	Univariate analysis (OS)			Univariate analysis (DFS)			Multivariate analysis (OS)		
	Hazard ratio	95 % CI	P*	Hazard ratio	95 % CI	P*	Hazard ratio	95 % CI	*P* ^†^
*Axl* mRNA expression									
Low	1		**0.047**	1		0.094	1		**0.04**
High	2	1.01–4.8		1.7	0.91–3.5		1.9	1.03–3.5	
Age (yr)									
<65	1		0.47	1		0.75	1		0.2
≥65	0.77	0.38–1.6		0.91	0.5–1.7		0.71	0.43–1.2	
Gender									
Male	1		**0.0045**	1		0.087	1		0.99
Female	0.3	0.17–0.73		0.57	0.32–1.08		1	0.43–2.1	
Smoking status									
Never smoked	1		**0.0026**	1		0.051	1		0.37
Smoker (ex or current)	4.3	1.5–6.4		2	1–3.5		0.68	0.28–1.6	
Tumor differentiation									
Well	1			1			1		
Moderately	4.5	1.4–9.8	**0.01**	2.6	1.2–5.2	**0.012**	1.3	0.73–2.2	0.39
Poorly	11.8	5–38.7	**<0.001**	4	2–11.8	**<0.001**	3	1.4–6.4	**0.004**
P-stage									
I	1			1			1		
II	0.55	0.12–3.1	0.56	1.3	0.41–4.6	0.6	0.63	0.22–1.5	0.33
III	5.9	4.6–26	**<0.001**	5.3	5.3–26.7	**<0.001**	2.1	1.1–4.1	**0.022**
Adjuvant chemotherapy									
No	1			1			1		
Yes	0.51	0.2–1.04	0.061	0.62	0.29–1.2	0.14	0.45	0.25–0.8	**0.0067**
*EGFR* gene mutation status									
Wild-type	1		**0.0077**	1		0.29	1		1
Mutation (+)	0.35	0.19–0.77		0.72	0.39–1.3		1	0.54–1.9	
*K*-*ras* gene mutation status									
Wild-type	1		0.23	1		0.12	1		0.63
Mutation (+)	0.49	0.23–1.4		0.46	0.25–1.2		1.2	0.55–2.6	

Bold values are statistically significant

*OS* overall survival; *DFS* disease-free survival; *CI* confidence interval

* Log-rank test

^†^Cox’s proportional hazard model

## Discussion

In a previous report, Wimmel et al.^16^ analyzed Axl and Gas6 expression in NSCLC and small cell lung cancer (SCLC) cell lines, and Shieh et al.^17^ reported correlations of Axl protein expression with LN involvement and clinical stage. Our findings on the impact of Axl expression on patient survival further corroborated these observations. Furthermore, our study is the first to identify *Axl* mRNA expression as a possible independent factor on clinical outcomes in lung adenocarcinoma.

In this report, we found that pleural invasiveness was specifically related to higher *Axl* mRNA expression, suggesting its pivotal role in tumor invasion, as revealed by Tai et al.^21^ Factors, such as p-stage and LN metastatic status, also were shown to be statistically associated with Axl protein expression, which is consistent with its essential role in epithelial-to-mesenchymal transition.^22^ The independency of *EGFR* gene mutation in relation to Axl expression also was revealed, which is surprising considering the possible cross-talk or alternative activation between Axl and EGFR.^23^,^24^ *K*-*ras* gene mutation, which is another important mutational pattern in NSCLC, also was found not to be correlated with either Axl or Gas6 expression level.^25^ Higher tumor Gas6 protein expression was shown to be related to not only advanced tumor status but poorer patient survival, suggesting its significance in tumor advancement.^11^

In contrast to these results, it was intriguingly shown that genetic expression of Gas6 had an opposite tendency than that of Axl and Gas6 protein expression. Irrespective of the fact that Gas6-dependent Axl phosphorylation results in subsequent activation of oncogenic pathways,^26^ Cormack et al. 27 revealed that increased *Gas6* mRNA expression was associated with favorable prognostic factors in breast cancer, and this phenomenon was also found in RCC.^10^ All of these aforementioned results seen in other types of tumors are concordant with the findings of our present study. Although the precise mechanism underlying this expression inconsistency has yet to be elucidated, it is postulated that while *Gas6* mRNA is naturally of tumor origin, Gas6 protein as detected by IHC is generally exogenous. It is already known that Gas6 is expressed not only by tumor cells but also by endothelial cells, fibroblasts, vascular smooth muscle cells, and infiltrating immune cells,^28^ and tumor cells are stimulated via paracrine as well as autocrine fashion by Gas6, which also is found in serum.^10^ It also is assumed that paracrine-derived Gas6 incorporation into tumor cells represses tumoral Gas6 expression per se, resulting in downregulation of *Gas6* mRNA expression. We hope that further investigations will clarify the underlying mechanisms.

In light of the limited therapeutic options currently available for NSCLC, the Axl/Gas6 system remains an attractive therapeutic target,^29^ and some small molecules with Axl inhibitory effects are already under development.^30^ Elucidation of the exact mechanisms of Gas6 stimulation on tumor tissue (i.e., autocrine and/or paracrine) would increase the efficacy of targeting this RTK axis for the treatment of lung adenocarcinoma.

In conclusion, we found that Axl and Gas6 were highly expressed in surgically treated lung adenocarcinoma tissues at both genetic and protein levels, revealing their marked correlations with clinical outcomes. We believe that our findings contribute to further understanding of these promising molecules in utilizing them as novel biomarkers and therapeutic targets in NSCLC.
